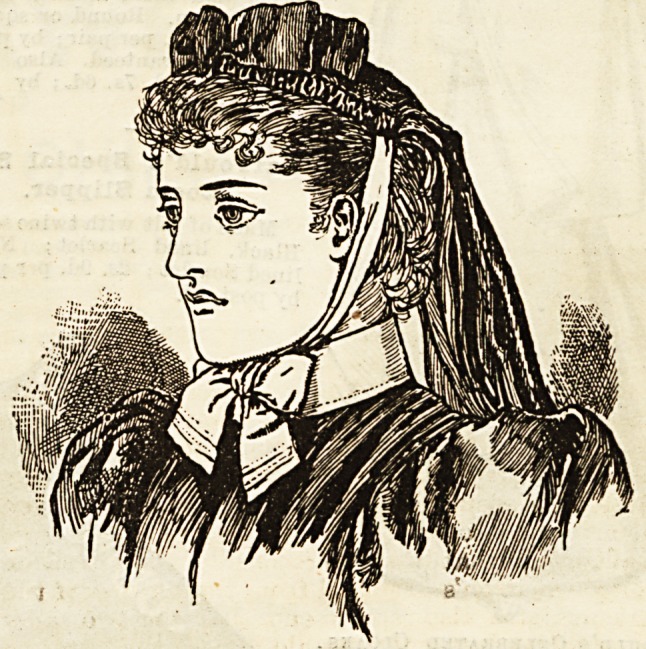# The Hospital Nursing Supplement

**Published:** 1895-03-02

**Authors:** 


"The Hospital" Nursing Mirror.

Being the Extra Nursing Supplement of "The Hospital" Newspaper.

[Contributions for this Supplement should be addressed to the Editor, The Hospital, 428, Strand, London, W.C., and should have the word "Nursing" plainly written in left-hand top corner of the envelope.]

### News from the Nursing World.

### LECTURES AT ST. MARY'S.

At St. Mary's Hospital, Paddington, of which H.R.H. the Duke of York is President, three courses of lectures are given to the nursing staff each year. The first twelve, just completed, were by Dr. Maguire on "The Elements of Nursing." Those now in progress, on "Elementary Anatomy and Physiology and Surgical Nursing," are by Mr. Silcock, and when they are finished Dr. Gow will give six lectures on "Obstetric Nursing." For a very moderate fee ladies not on the nursing staff of St. Mary's Hospital are admitted to these lectures.

### LEWISHAM INFIRMARY.

At the meeting of the Lewisham Guardians last week a long discussion took place on the presentation of a report by a committee, advising that the assistant matrons, Miss Mills and Miss Campbell, should be called upon to resign. They and other members of the nursing staff had addressed a communication to the Local Government Board respecting the reinstating of Miss Patterson; also denying the truth of the assertion recently made as to harmony having prevailed since that matron's suspension. Extracts from this letter were handed to the Guardians by the Local Government Board, and the Infirmary Committee have reported upon it. Considering it reflected on their previous action, and "revealed a wilful spirit of insubordination," they desire the departure of the two assistant matrons. A suggestion that an enquiry might be made before so strong a measure was taken, did not receive encouragement. The matrons, however, reserved their reply.

### DR. JAMES ANDERSON.

Many of our readers will be interested to hear that the committee of the Dr. James Anderson Memorial have received £300 to invest. This was announced at the Aberdeen University Court meeting by Dr. Alexander MacGregor, and it has been agreed to make an annual award to the best student in clinical medicine at Aberdeen University. The examination will be conducted by Professor Finlay and Dr. Angus Fraser at the end of the winter session, the first taking place in the current month. The award will consist of a gold medal (not to exceed £7 in value), the balance of the interest of the fund being given in the form of books, instruments, and money to the successful student.

### NURSE AND CARETAKER.

At present the Warminster Infectious Hospital seems to be empty, and, according to the local press report, there is a difficulty in finding a nurse for it. Her duties will include those of a caretaker, and the combination of offices is rewarded only by free quarters, and, when there are patients, a shilling a day is paid for the first, and sixpence a day for each of the other cases admitted. This rate of remuneration does not seem to commend itself to nurses of the present day, although it is asserted that hitherto competent persons have been found willing to accept it.

### PRIVATE NURSES AT LEEDS.

The nineteenth annual meeting of the Leeds Trained Nurses' Institution was held last week at the Home and a most satisfactory financial and general report was presented. One hundred members of the staff are engaged in private nursing, and seventeen are receiving training in various hospitals. Rest and change are, when necessary, provided at the Woodlam Home, Headingley, and an annual subscription paid by the society to the Royal National Consumption Hospital at Ventnor secures admission for any nurse for whom a visit there is judged desirable. Another proof of the consideration of the committee for the nurses is shown by their again paying £100 into the Leeds Trained Nurses' Institution Trust Fund of the Royal National Pension Fund for Nurses. This example might well be imitated by other institutions.

### WOOLTON.

The Woolton District Nursing Society has issued its sixteenth annual report, showing 6,833 visits have been paid to 223 patients during the year. Nurse Key appears to enjoy the thorough confidence of the sick poor, and her untiring labours in their behalf are appreciated by her committee. The rules of the society seem good, and the trained nurse which it secures to the village and district of Woolton is expected to teach nursing, domestic economy, order, cleanliness, and ventilation, in addition to personally attending the sick.

### AN EXCELLENT EXAMPLE.

It was pleasant to note in reports of a recent Board meeting of the Dover Guardians how promptly the humane suggestions of the chairman were followed; improved and varied diets for the children being sanctioned, and also certain additions to the nursing staff. The engagement of two more nurses will secure proper night attendants for the sick and infirm, who have had hitherto to depend on pauper help unless they disturbed the day nurses in their well-earned rest. The action of the Dover Guardians may well be commended to the notice of those at Coventry, who, in spite of repeated warnings from the Local Government Board, remain still unconvinced on the subject of night nursing. At a meeting held lately one-half of the Guardians present voted against the introduction of a night nurse in their infirmary, regardless of the representations of those favourable to "the dictates of common sense and common humanity."

### GOOD MANAGEMENT.

The Private Nursing Department of the Kent and Canterbury Institute for Trained Nurses is now self-supporting, and appears, from the annual report, to be working satisfactorily. Only four nurses have as yet joined the Royal National Pension Fund, but it is hoped and expected that a greater number will do so in the future. The services of the district nurses have been much appreciated during the year, and this branch of the work is entirely charitable, being maintained by donations and subscriptions. A recently purchased house, with a garden, will conveniently accommodate the whole staff, which has been hitherto somewhat cramped for room. The committee express much satisfaction with the excellent management of the lady superintendent, Miss Shaw.

### NURSES AT NORTHAMPTON.

The eighteenth annual report of the Northamptonshire Institute for Trained Nurses shows that several important alterations have taken place there. At the suggestion of the lady superintendent, Miss Wathen, one of the houses formerly occupied by the society has been relinquished and the remaining two constitute a more compact and convenient Home. Twelve private and three district Queen's nurses find ample accommodation, and one room is set apart for in-patients. There are five probationers undergoing two years' training, either at the Royal Infirmary, Edinburgh, or at Worcester or Peterborough; the shorter term named in the report is discountenanced, and candidates are only in exceptional cases taken under twenty-four years of age. The work of the Queen's nurses has been most satisfactory during the year, and the value of their services appears to have been fully recognised by the sick poor.

### A HOMŒOPATHIC HOSPITAL.

At the annual meeting of the Devon and Cornwall Homœopathic Hospital thanks were unanimously accorded to the hon. medical staff and to the nurses. It was decided that application should be made to the Chief Constable at Plymouth that accidents occurring in its immediate neighbourhood should be sent into the hospital.

### A GIRL-NURSE.

The Medical Officer at Ballymena Union has brought the need for the appointment of a night nurse forcibly before the Board. At a recent meeting considerable diversity of opinion as to this necessity appeared to prevail, some of the Guardians, according to the local press, considering an addition to the present staff quite superfluous. Others, however, comprehended the duty of providing proper care for the sick and infirm during the night. Eventually the matter was postponed for consideration at the next Board meeting, and before that takes place we trust the Guardians will see the obvious impropriety of engaging "as probationary nurse a girl of eighteen or nineteen." She might, indeed, be possessed of a taste for nursing, and accept the £8 or £10 per annum suggested, but she would certainly be too young for work in the sick wards of a workhouse.

### THE PRESIDENT AND THE HOSPITALS.

President Faure is continuing his inspection of the Parisian hospitals, both Lariboisière and St. Martin's being honoured by visits on the 21st ultimo. All the wards of the former were entered in turn, those in the infectious block not being omitted. The president left a donation on concluding an inspection which commenced at nine a.m. St. Martin's is a military hospital, and the kitchens as well as the wards were visited, and M. Faure commended the quality of the beef tea and wine, both of which he appears to have tasted. This systematic inspection of hospitals carried on from week to week by the President is viewed with cordial approval by the Parisians.

### ITALY IN MAY.

We learn from the secretary of the Goole Cottage Hospital that a party is being organised to visit Italy in May. The railway companies have offered advantageous terms if the number is made up to thirty. It is, therefore, estimated that the cost to each person will not exceed thirteen guineas. May 18th is the date fixed for starting, and the expedition will take fifteen or sixteen days. First-class accommodation will be secured at hotels and second-class tickets for the journey, which is arranged so as to give six days at Venice, one at Verona, two at Milan, one at Lucerne, and three or four amongst the Italian lakes. Mr. Wells, secretary, Goole Cottage Hospital, will be pleased to give full particulars, or to receive applications from any one desiring to join the party, which is not yet completely made up.

### NURSES AT VANCOUVER.

The Report of the work at St. Luke's Home, Vancouver, last year, does not include any balance-sheet of income and expenditure, although a few donations are published. The patients nursed in the Home numbered 58, and 37 were tended outside. Of these cases 23 were free, and many of the others have, owing to the prevalence of "bad times," paid only a portion of the fees due to the Home; therefore, the Sister in Charge pleads for increased financial support. Two of the nurses contracted diphtheria from two patients at Vernon towards the close of the year. Strict isolation was enforced, and the four cases were treated in cottages set apart for the purpose, the mayor and city council providing all that was necessary, as well as guards night and day, to ensure the quarantine being respected. Sister Frances reports that Christmas at St. Luke's was unusually quiet, the customary entertainment being omitted in consequence of the recent death of a nurse lately arrived from Toronto, and also on account of the serious illness of another of the workers.

### A PARISIAN MATERNITY CHARITY.

Patients at the "Mutualité Maternelle," Paris, have for a month after their confinement an allowance equivalent to the money they might have earned by their work during that period. A weekly subscription of 2½d. secures to the mother medical attendance "at a nominal price," and, if she nurses her own child, she is presented with a gift of twenty francs.

### SHORT ITEMS.

The Settle and District Nursing Association has completed a fourth year's useful work.—The first year's work of the Filey district nursing has been satisfactory, and a balance in hand is reported.—At the annual meeting of the Egham Trained Nurses' Association a report of its excellent work was laid before the subscribers.—A sacred concert at Hebden Bridge resulted in £19 17s. being handed to the local nursing institution.—The ball given on February 12th at the Queen's Hall, Langham Place, in aid of the funds of the Great Northern Central Hospital was a brilliant success, resulting in over £200 being handed to the hospital.—Miss Tilbury has resigned the position of superintendent of nurses at Lambeth Infirmary.—A jumble sale was carried out in aid of the Ballymena District Nursing Society on February 14th.

### Elementary Anatomy and Surgery for Nurses.

By W. McADAM ECCLES, M.B., M.S., F.R.C.S., Lecturer to Nurses, West London Hospital, &c.

### VII.—THE PRINCIPAL JOINTS OF THE BODY; WITH THE CHIEF MUSCLES WHICH MOVE THEM.—*continued.*

### THE JOINTS OF THE LOWER EXTREMITY.

(1) Each os innominatum is attached to the sacrum, and to its fellow by the variety of articulation known as a

FIG. 14.—The Pelvic and Hip-joints.

yielding joint. (2) The hip joint (see Fig. 14) is a most perfect ball-and-socket articulation, the head of the femur fitting very accurately into the acetabulum of the os innominatum. The chief ligaments are the capsular, strengthened by certain accessory bands, and within it the cotyloid ligament

FIG. 15A.—Front view of the Knee-joint.

FIG. 15B.—The interior of the right Knee-joint.

around the margin of the acetabular cavity, the notch at the lower part being filled by the transverse ligament stretching across, and lastly, the so-called round ligament attached to the depression on the head of the femur and the bottom of the socket. (3) The knee-joint (see Fig. 15A and 15B) is formed by the contact of the two condyles of the femur above, the expanded head of the tibia below, and the posterior surface of the patella in front. There are numerous ligaments holding the bones together—anterior, posterior, and lateral practically forming a capsular, and in the interior of the joint two cruciform ligaments, and two semilunar interarticular fibro-cartilages which act as buffers when we jump. The fibula is connected with the upper end of the tibia by a joint. (4) The ankle joint (see Fig. 16) is a hinge joint, the bones of which are the tibia above, with its inner malleolus internally, and the outer malleolus of the fibula externally. In the arch thus formed fits the astragalus.

The joint between the lower jaw and the temperal bone on either side is worthy of mention. It has an interarticular

FIG. 16.—Inner view of the Ankle-joint.

fibro-cartilage. (See Fig. 17.) The several vertebræ are connected together by yielding joints, thus making the spinal column very pliable.

Muscles constitute what is ordinariiy spoken of as the flesh and clothe the bones, forming the bulk of the soft parts. They are red from the presence of a large number of vessels containing blood. Their attachment to bone is usually by fibrous tissue termed tendon, and seen as the leaders. The attachment to the least movable part is called the origin of a muscle, while its insertion is to that part which is to be moved. The chief muscles which it will be necessary here to allude to are certain of those moving the bones which enter into the joints of the

FIG. 17.—The Temporo-maxillary joint.

limbs above described. These may be conveniently for the most part arranged into two groups: (1) The flexors which bend joints, and (2) the extensors which straighten them. The two important muscles about the shoulder joint are the deltoid which forms the rounded outline of the shoulder, and raises the humerus to a right angle with the body; and the pectoralis major the large breast muscle which draws the arm down again to the side. The elbow is flexed by the biceps chiefly, and extended by the triceps which lies behind the arm. The fingers have a large number of special muscles going to them, whereby the delicate movements so necessary are satisfactorily performed. When the hand lies on the table with the palm downwards it is said to be pronated, but becomes supinated when the palm lies upwards. Special muscles, the pronators and supinators, bring about these movements. The hip joint is flexed by a large and powerful muscle which arises from the front part of the lumbar vertebræ within the abdomen, and also from the iliac bone; this is known as the ilio-psoas muscle. It is extended by the gluteus maximus, the great muscle constituting the prominence of the buttock.

The knee is bent by the hamstring muscles lying behind the thigh, and straightened by a four-headed muscle, the quadriceps extensor, which is inserted into the patella.

The toes are pointed by the calf muscles uniting for the most

Fig. 18.—The Diaphragm.

part in the tendo Achillis, which also raises the heel. They are drawn up by the muscles passing down from the anterior part of the leg. There are numerous short muscles in the sole of the foot which aid in maintaining the longitudinal arch, and help to give elasticity to the foot.

The diaphragm is the muscular partition of an arched form between the thorax and abdomen. It is an important muscle of respiration. (See Fig. 18.)

### Appointments.

[It is requested that successful candidates will send a copy of their applications and testimonials, with date of election, to The Editor, The Lodge, Porchester Square, W.]

Warneford Hospital, Leamington.—Miss Katherine Rapson has been appointed Matron of this hospital. She was trained at Westminster Hospital; was sister at Greenwich Hospital, and, for the past four years, has been assistant-superintendent of the General Infirmary, Leeds. We congratulate Miss Rapson on her new appointment, and wish her every success in it.

Ramsay Hospital, Naini Tal.—Miss Rundle has been appointed Matron-Superintendent of the Ramsay Hospital, Naini Tal, in place of Miss Wildman, who resigned. Miss Rundle was trained for one year at University College Hospital. During some years past she has been private nursing in India, and for a short time assisted in Lady Roberts' Hospital for Sick Officers, at Murree.

Wallasey Cottage Hospital.—Miss Alys Barry has been made Matron of this hospital. She received three years' training at the Leicester Infirmary, and was Ward Sister for nearly two years at the Birmingham Workhouse Infirmary. Miss Barry has our sincere good wishes for success in her new work.

Blackburn and East Lancashire Infirmary.—Miss Henrietta C. Poole has received the appointment of Matron at this infirmary. She was trained at St. Bartholomew's Hospital, and has been Superintendent of Nurses and Matron at the Adelaide Hospital, Dublin. We congratulate Miss Poole on her new appointment.

### Nursing in New South Wales.

### PRINCE ALFRED HOSPITAL, SYDNEY.

#### The Nurses.

It is more than two years since the fine Nurses' Home was completed and occupied, and everything connected with it has added greatly to the comfort of the staff. Miss S. McGahey's administrative abilities are exceptionally excellent, and all her efforts for the advancement of trained nursing in New South Wales have received cordial support from the hospital committee, with the result that the Prince Alfred Training School at Sydney is second to none. Not only is the work entirely satisfactory, but a general tone of kindly courtesy is characteristic of this admirable school. Due appreciation of the training is shown by the frequent appointments bestowed at other hospitals on nurses from the Prince Alfred. During the last six or seven months, for instance, one sister has left to take the post of matron at the Napier Hospital, New Zealand; a nurse from Prince Alfred Hospital has entered the same hospital as charge nurse, whilst another has gone to the Auckland Hospital as night superintendent. Three other nurses trained at the Prince Alfred have received appointments as matrons of the Parramatta District Hospital, the Western Suburb Hospital, and Kagurali Hospital respectively.

It may be interesting to English readers of The Hospital to learn that the grades in which the probationers and nurses are classed at this Colonial training school, are as follows: 1, Probationers; 2, Junior Nurses; 3, Nurses; 4, Ward Nurses; 5, Charge Nurses. When probationers have passed their first examination and completed their first year's training to the satisfaction of the committee, they are eligible for promotion, at the discretion of the committee, to the position of junior nurses, *i.e.*, when vacancies occur.

When junior nurses have passed their second examination and completed their second year's training to the satisfaction of the committee, they are eligible for promotion to the position of nurses as vacancies occur. When nurses have passed their third year's examination, and completed their third year's training, if found thoroughly competent in every respect, they will be eligible for posts as ward nurses. Ward nurses are selected from those who have received certificates.

Charge nurses selected from the ward nurses have complete charge of two small wards each containing twelve beds.

Certificates are given to nurses who pass through their three examinations and their three years' training to the satisfaction of the committee. Certificated nurses who wish to remain on the staff can do so for six months (after receiving their certificates) at the discretion of the committee, but those who have not been promoted to be ward nurses by that time must then retire.

The sisters are chosen from the charge nurses. The number of women who undertake private nursing in Australia without proper training, and the indiscriminate adoption of the title of nurse by those who have no right to it, disastrously affect the prospects of duly trained nurses in the colonies. The length and the quality of the training provided at the Prince Alfred Hospital is of the very first order, and yet nurses on leaving may find themselves competing for employment with persons of little or no qualifications. The evil has become so universally recognized that a petition signed by over fifty trained nurses, including matrons and sisters, has been sent to the British Medical Association of New South Wales asking for help and advice with regard to the "Trained Nurse Problem."

### Where to Go.

The Sailors' Bazaar is to be opened at the Cannon Street Hotel, March 26th, by the Duchess of Fife. It is in aid of the British and Foreign Sailors' Society.

Clapham Maternity Hospital, 41, Jeffrey's Road, S.W.—The annual meeting will be held on Friday, March 1st, at three p m.

### Troubles at Lewisham Infirmary.

In the The Nursing Mirror of February 9th we mentioned the results of the Local Government Board inquiry at Lewisham. Their recommendation for the reinstatement of the matron at the infirmary did not meet with the approval of the Guardians, who sent a deputation to the Local Government Board on the subject, as we noted in our issue of February 16th. It was advised at this interview that one of the members should see the matron, but this suggestion was not carried out.

The following extracts from the communication addressed to the Local Government Board by members of the nursing staff at Lewisham Infirmary are from the report in the local press:—

We the nursing staff of the Lewisham Union Infirmary desire to express our pleasure and satisfaction at the result of the inquiry recently held here by Dr. Downes, as regards the reinstatement of our matron, Miss Patteson; and we earnestly beg the Local Government Board not to reconsider their decision, as suggested by the Lewisham Board of Guardians, but that an early date might be fixed by the Local Government Board for her to resume office. We would also express our strong disapproval of the statement made at the meeting of the Board of Guardians on February 4th, to the effect "that peace and harmony now existed in the infirmary, whereas before all was discord." We desire to contradict that statement, and to say that the last twelve weeks, since the suspension of the matron, has been a time of great discomfort and discord to us all, and the relations between officials most strained, and we have many of us been subjected to things, both said and done, of an extremely unpleasant character. We have been kept working together in our various positions by our sense of duty and a feeling of loyal respect for our matron, hoping and believing that she would be reinstated, and that our grievances would then receive attention and matters placed on a better and pleasanter footing. We beg to mention one grievance which we feel keenly, and which we have reported in a letter to the chairman of the Board of Guardians (a copy of which we enclose) on December 4th, 1894. To this we have as yet received no answer, the grievance being the bringing into force of a new time table drawn up by the Guardians, instead of the one which we agreed to when we were elected. Our objection is stated in the letter. We desire, in conclusion, to express our entire confidence in our matron and our admiration of the forbearance she has shown under great provocation. We most respectfully beg that this petition may receive your consideration at an early date, and that the matron may receive the support of the Local Government Board in face of the strong opposition and animosity displayed against her.

(Here follow 18 signatures.)

Upon this communication the Infirmary Committee reported as follows:—

The committee are exceedingly surprised that a body of trained officers should so far forget themselves and their position as servants of the Board of Guardians that they should set the Board in open defiance, and by their conduct reveal a spirit of wilful insubordination as existing among such a large proportion of the officers of the establishment, who practically charge the Guardians with displaying strong opposition and animosity towards the matron. The committee cannot but feel that great pressure must have been brought to bear upon the nurses in signing the document which was transmitted to the Local Government Board. That that Board be informed in reply to their letter to the effect above expressed; also that the circumstances connected with the letter referred to appear to fully justify the Guardians' action in suspending the matron, and that they adhere to their former action in the matter, and they urge upon the Local Government Board to reconsider their decision with a view to the matron's dismissal from the service of the Board. The committee also recommend that the two assistant matrons, at whose instance it would appear the letter to the Local Government Board was originated, be called upon to resign their appointments; but before adopting this suggestion that they be asked to attend before the Guardians in explanation of their conduct.

The assistant matrons attended as requested, and when informed of the resolution of the Board they, at the suggestion of the chairman, reserved their reply.

### Burdett's Official Nursing Directory.

### A DIRECTORY, NOT A REGISTER.

It is well to bear in mind the difference and distinction which exists between a register and a directory. As to the propriety or otherwise of instituting registration of nurses there are various opinions for. A register implies rights, and suggests that those who are not upon it are in some way or another inferior beings. But a directory is another matter. A directory gives no rights; insertion in it implies no adherence to any particular party or any particular opinion, and in no way interferes with entry on the register of any association, or the list of any institute, to which the individual may belong. All that a directory professes to do is to give information. The "Medical Register" is not a mere list of qualified medical men; it is admission to the Register, which itself makes him qualified. For years, perhaps even now, "Churchill's Medical Directory" contained the names of medical men who, so far as public appointments were concerned, were unqualified; it also contains the names of homœopathists, whom four out of every five medical men will not meet; and it certainly contains the names of many for whose moral character we should not like to vouch. But it gives full and accurate information about them all, and for the sake of that information it is constantly consulted both by the public and the doctors. At present there is no Register for the nursing profession giving any legal status. Such a thing may come or it may not; but in either case there is no reason why there should not be a Directory, giving such information about the career of each nurse as both the public and the profession want; information, by the by, which no legal Register would be likely to insert. The "Medical Register" is a bald and meagre thing compared with the "Medical Directory." What Burdett's "Official Nursing Directory" proposes to do is to supply the want of a directory giving information. It will give no rights, it will imply no partisanship, it will give no guarantee except that what it prints is true, and is derived from official sources. This ought to be enough to make it acceptable to the public, and every nurse ought to make sure that, whatever list or register she may be on, her name shall also appear on Burdett's "Official Directory," for we understand that already applications for admission to it are crowding in so fast as to be difficult to deal with, showing how rapidly it is spreading in public favour, and pointing to the certainty of its success.

### Minor Appointments.

Old Somerset Hospital, Capetown, South Africa.—Miss Emily Eighleen, who was trained at the London Hospital, and after gaining her certificate held the post of Staff Nurse Queen, has recently been appointed Charge Nurse in the new wards at Old Somerset Hospital. A similar appointment has been conferred on Miss Phyllis Cooper, who, after gaining her certificate at the London Hospital, became Staff Nurse Mary, and afterwards joined the private nursing staff. Both nurses take with them many cordial wishes for success in the new work to which they start in the course of this month.

### Death in Our Ranks.

Florence Sarah Hole, aged 23, a nurse in the Salford Royal Hospital, died from typhoid fever at the hospital on the 15th inst. She joined the staff as probationer in April, 1894. Her loss is deeply felt by the staff of the hospital. Her gentle and pleasant manner made her a great favourite with all.

### Everybody's Opinion.

[Correspondence on all subjects is invited, but we cannot in any way be responsible for the opinions expressed by our correspondents. No communications can be entertained if the name and address of the correspondent is not given, or unless one side of the paper only be written on.]

### OUR PRINCE AND PRINCESS.

"Scottie" writes from Edinburgh: You have done a good work in producing the splendid portrait of "Our Princess," a work for which many thousands of your readers are grateful. I have it framed and hung up in the place of honour in my best room, and all who have seen it declare it looks lovely but lonely. I would, like many others, be delighted if you would publish a companion portrait of Our Prince, H.R.H. the Prince of Wales. Willingly would I place my name on a subscription list for a guinea if you would publish it that way, rather than have "Our Princess" alone, it does not seem natural. They are together in all good works; they are together in our thoughts and affections, and we would be glad to have their portraits together in our homes. Our Prince is a friend and brother to us all, rich and poor alike, and deserving of all the respect, honour, love, and loyalty we can give him. Wherever there is a good and charitable work to be commenced, there he is, trowel in hand, ready to begin, support and carry it on; he is a prince we are proud of, and none love him more truly than the sick poor and afflicted, to whom he has always been such a good friend and patron. If we cannot have himself up here in the North half as often as we wish, do please let us have his portrait, similar to that of our bonnie Princess, so that we may have them together in our homes as in our hearts. As a true and loyal subject of our gracious Queen, I earnestly trust and hope they may continue to do good hand in hand together, in their present station, for very many long and happy years to come.

*** All in good time. Christmas, and with it the Christmas number, comes but once a year.—Ed. *T. H.*

### GREETINGS FROM INDIA TO "OUR PRINCESS."

A correspondent writes: I was much pleased with the Christmas number of The Hospital you sent me, especially with the lovely and beautifully finished portrait of our Princess. Only a few weeks ago I was regretting so much that, though a loyal subject and servant of the Queen-Empress, no portrait of either Her Gracious Majesty or any members of the Royal Family adorned my drawing-room. I hunted the shops and the bazaars, but the portraits that were to be obtained there were such travesties of the originals that I preferred to carry rather the memory of those God has placed over us in the Far West. Our Indian rulers—the late Commander-in-Chief, Sir George White, our present Chief, and also our Principal Medical Officer — are well represented, and now, as if in answer to my wish, comes the portrait of our fair Princess. I have had it cut off the thin white mount, and carefully mounted by our local photographer on one of those cards that are in vogue for mounting platino types; that with the lithographed autograph carefully affixed, framed in a handsome carved Indian frame, makes a valuable and artistic addition to my portrait gallery, and I shall wait in hope for an enterprising editor to send me next year an equally charming one of Her Gracious Majesty. Christmas makes little or no difference to us out here, and makes but little break to our sick in the even monotony of their days. One of the tradespeople on Christmas Eve sent me a hamper of mistletoe and holly. Some kind ladies sent Christmas cards, about three a head. I tied up the holly and mistletoe into small bunches, and in the dead of night, when all was quiet and patients asleep, the orderlies and I silently tied these emblems of Christmas in the homeland over each bed, and put the cards on the bedside tables. You good people over the *Kali pani* can not, amidst your Christmas luxuriance, possibly imagine one-hundredth part of the pleasure those simple decorations gave to those who had not seen holly and mistletoe for years. Tears came into some of their eyes, and one poor man said to me, "If I had only known old Santa Claus was coming round last night, I'd have lain awake to wish him a happy Christmas." The men were like so many children over their Christmas cards and their decorations.

### THE SOCIETY OF TRAINED MASSEUSES.

Mrs. Arthur writes: I saw an allusion last week in The Hospital to the Society of Trained Masseuses, so beg to forward you the following circular. An examination has just been held, and the next one will be in April.

The Society of Trained Masseuses has been formed for the purpose of improving the training of, and organising an independent examination for, competent masseuses. It is hoped this may establish a more uniform standard of proficiency and qualification. The Council holds periodical examinations for candidates, who are required to produce satisfactory evidence of training and moral character. Candidates will be examined in the theory and practice of massage by two examiners other than their own instructors. Notice of the examinations will be given in *Nursing Notes*. Successful candidates, after signing the required undertaking, will receive the formal certificate of the society, and will then be entitled to have their names placed on its roll. Further particulars can be obtained on application to Mrs. Arthur, hon. secretary, Society Trained Masseuses, 12, Buckingham Street, Strand, London.

The Council: Miss Buckworth, Miss Griffiths, Miss Manley, Miss G. Manley, Miss Molony, Mrs. Palmer, Miss Robinson, and (hon. secretaries) Mrs. Arthur and a Member of the Council Trained Nurses' Club, *ex officio*. Rules of the society (signed by each successful candidate): 1. No massage to be undertaken except under medical direction. No general massage for men to be undertaken. Occasional exceptions may be made at a doctor's special request for urgent or nursing cases. 2. No advertising permitted in any but strictly professional papers. 3. No sale of drugs to patients allowed.

### Notes and Queries.

### Queries.

(86) *Insurance.*—Where can I insure against accident or illness?—*Medical Nurse.*

(87) *Ambulance.*—You can get all particulars of the St. John Ambulance Association.—*Wanted.*

(88) *Male Nurses.*—I want to know all about the training of male nurses, and shall be glad of full information on the subject?—*Candidate.*

(89) *Training.*—Where could a lady obtain training in the nursing of heart cases and paralysis?—*Helen.*

### Answers.

(86) *Insurance* (*Medical Nurse*).—You can make provision for yourself in such cases by joining the Benevolent Branch of the Royal National Pension Fund. You should write for particulars or call on the secretary at 28, Finsbury Pavement, London, E.C.

(87) *Ambulance* (*Wanted*).—By writing to the secretary of the association at St. John's Gate, Clerkenwell, London, E.C.

(88) *Male Nurses* (*Candidate*).—In England no general hospitals train male nurses. We have often replied to queries on this subject in the "Nursing Mirror."

(89) *Training* (*Helen*).—In a workhouse infirmary, under a matron who is a fully-trained nurse, great experience in such cases is obtainable. You would also learn much in the medical wards of a large general hospital.

### Wants and Workers.

[The attention of correspondents is directed to the fact that "Helps in Sickness and to Health" (Scientific Press 428, Strand) will enable them promptly to find the most suitable accommodation for difficult or special cases.]

Votes for the May election at the Royal Hospital for Incurables, West Hill, Putney Heath, will be thankfully received by "Nursing," care of Editor The Hospital, 428, Strand, London.

Emily Baker has been an inmate of the Cottage Hospital, Lyme Regis, since May, 1893, suffering from spinal disease. For several months she had to lie in one position only, and nearly the whole period of her stay she has been unable even to read, or do any little thing to beguile the weary hours. She is now getting better, though very far from being recovered, and it is necessary she should leave the hospital, but she has no means of support, and her sisters cannot afford to contribute much. She is in every way a deserving person, and has borne her long illness with exemplary patience. I am trying to raise some money by small contributions so as to help her for a time, and would feel grateful for any donations. Contributions may be paid either to the Matron or the Hon. Secretary, Mr. R. W. Hillman, Lyme Regis.—Charlotte Egee, Matron.

### Asylum News and Nursing.

[Contributions to this section should be addressed to The Editor, 428, Strand, W.C., and have the words "Asylum News" written in left-hand bottom corner of the envelope.]

### LETTERS FROM AN ASYLUM NURSE.

An Asylum From Within.—The Commissioners' Visit.

I had not been many weeks at the asylum before a nurse from another ward rushed up to our charge nurse in a state of great excitement, and exclaimed "The Commissioners are here!" The reply was, "Drat them! I wanted my day off so badly, and now I shan't get it." As the lavatories were in my charge, I asked her if I should get them done up. "No," said she; "you can see that they're clean, but I'll have no Commissioners' soap in my ward. If it's good enough for the doctor, it's good enough for them!" She had not time to explain to me what she meant by "Commissioners' soap," but I found out afterwards. The term arose from nurses, over-anxious to make a good impression, placing newly-cut bits of soap in the dishes, and thus giving rise to the idea that it had only been put there for show. All that seemed to concern her was whether the nurse in charge of the dormitory, a young woman who required sharp looking after, had been neglecting her work that morning. "I couldn't get up to see your beds this morning, but if they are not all right you'll catch it."

Presently the matron came round and gave instructions that all the patients were to be seated, and as far as possible in alphabetical order. This gave us some trouble, and considerably disturbed certain of the women, who were always ready to make a bother. I was rather surprised at this order, because I thought the Commissioners would want to see the wards in their every-day condition. In a little while two elderly gentlemen, with the doctors and matron, came into the ward. One of them had a notebook in his hand, and as each patient's name was told him, he ticked it off from a list. He seemed a kind and sympathetic gentleman, but looked so wearied of his monotonous occupation that I really felt sorry for him. For a gentleman who must have attained a high position in his profession, it seemed to me a very useless and tedious employment. It was, of course, to ensure that every patient had an opportunity of speaking to him. The other gentleman walked about and inspected the ward and dormitories. From what I could gather, his business seemed to be chiefly to look out for something to find fault with. I am only a nurse, but it struck me that some of the things he complained of were of a very trivial character, and, indeed, sometimes purely accidental. The patients who did the greater part of the talking to the gentlemen were the most insane in the ward. After all the names had been ticked off the Commissioners went away.

"Is that all?" I asked the charge nurse.

"Oh, yes!" said she; "they will report upon our ward, and the way we take care of the people, from what they've seen."

"But," I said, "what can they possibly know about it, after a formal visit like this?"

"Young woman," returned she, "don't hurt yourself by thinking too much. Many people besides yourself have wondered that. What else could they do? Consider how many patients there are in this asylum, and how many asylums they have to visit."

"Well," I returned, "it must be very tiring work. Probably two gentlemen who have been medical superintendents, and accustomed to asylums, can form an opinion very rapidly."

"Very likely," snapped she; "but then one of them isn't a doctor at all, he's a lawyer."

"Then what can he know about it?"

"That's what we all want to know," she grumbled; "go about your work." The loss of her day off had upset her considerably.

### EFFECN OIN LUCOLD ON LUNATICS.

At one time very extraordinary ideas as to the power of the insane to resist cold were current. It was believed that an insane person could, with impunity, be subjected to cold and exposure which would be fatal to a sane person. Several explanations were advanced to account for this phenomenon. Among others, it was said that the circulation of the blood in lunatics was more rapid than in other people, or that the lunatic was in a state of continued fever. It is now known that the power of the lunatic to resist cold, instead of being greater, is less than that of a sane person; yet, so generally was the contrary believed at one time, that we find eminent writers thinking it necessary to disprove it. It is painful to read some of the regulations with regard to the care of lunatics in the beginning of the century. Thus, because the lunatics were peculiarly liable to "mortifications," arising from cold and confinement, it was expressly ordered that "every patient under strict confinement shall have his feet examined every morning and evening in the cold weather by the keeper, and also have them constantly wrapped in flannel." The impaired circulation and the diminished nerve-force of many of the lunatics explain their liability to suffer. It is not difficult to see how the misconception arose. The demented or the acutely-excited patient, unable to appreciate his sufferings, gave no sign that he felt the cold, though striking evidence of its effect might be readily found on examination; and, furthermore, the violent muscular exercise of the acute maniac helped him to withstand the cold to which his restlessness and destructive habits exposed him. The rise of temperature found in the majority of the latter class of cases will hardly justify the theory of continued fever in the ordinary sense of the term.

### Novelties for Nurses.

Nurses' Outfits.

We are glad to see that that enterprising house, Messrs. J. R. Roberts, of Stratford, are about to make a speciality of nurses' outfits. This will be a very great convenience to the large number of nurses in the neighbourhood to whom a long journey in search of the necessary requirements is often an impossibility or at least an inconvenience. The bonnet of which we give an illustration is a pretty little shape in fine black straw, fitting close to the head and bound round the brim with a quilling of black velvet. A daintily constructed bow of ribbon consisting of three loops is placed [illegible] front, and a long gossamer veil hangs behind. Wide ribbon strings tie in a neat bow under the chin. All the materials used are of the best quality and will wear well, the bonnet therefore is wonderfully cheap at 8s. 11d., which is the price at which it is quoted.

## Figures and Tables

**Fig. 14. f1:**
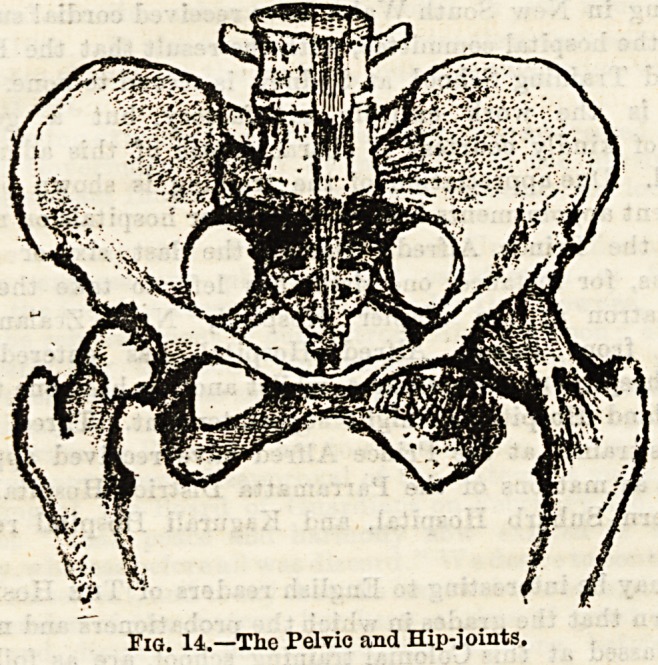


**Fig. 15a. f2:**
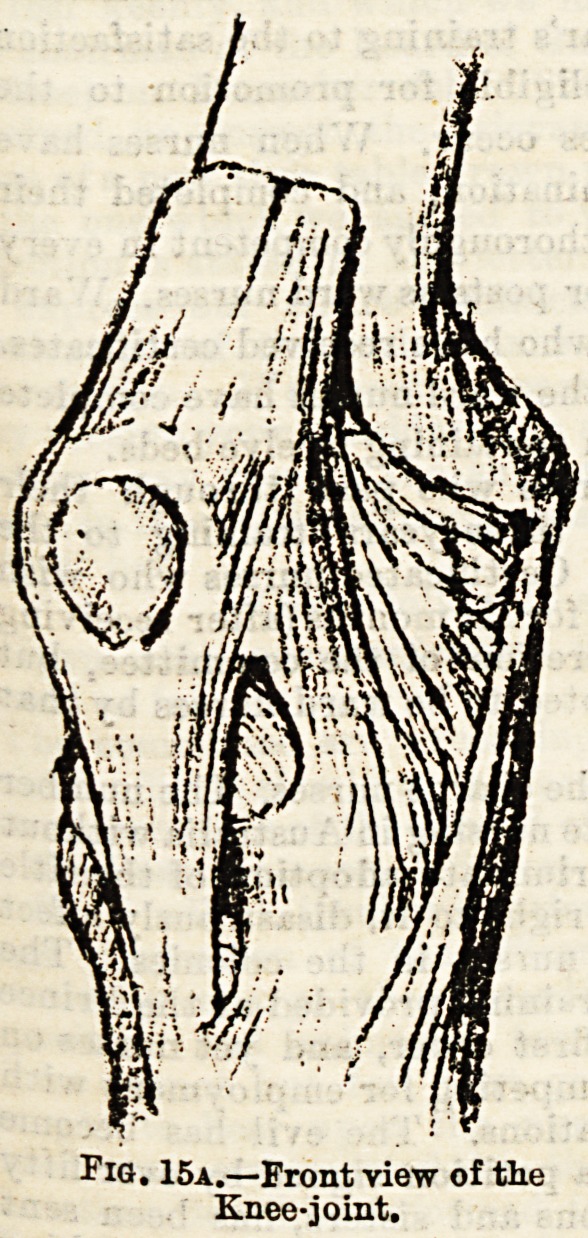


**Fig. 15b. f3:**
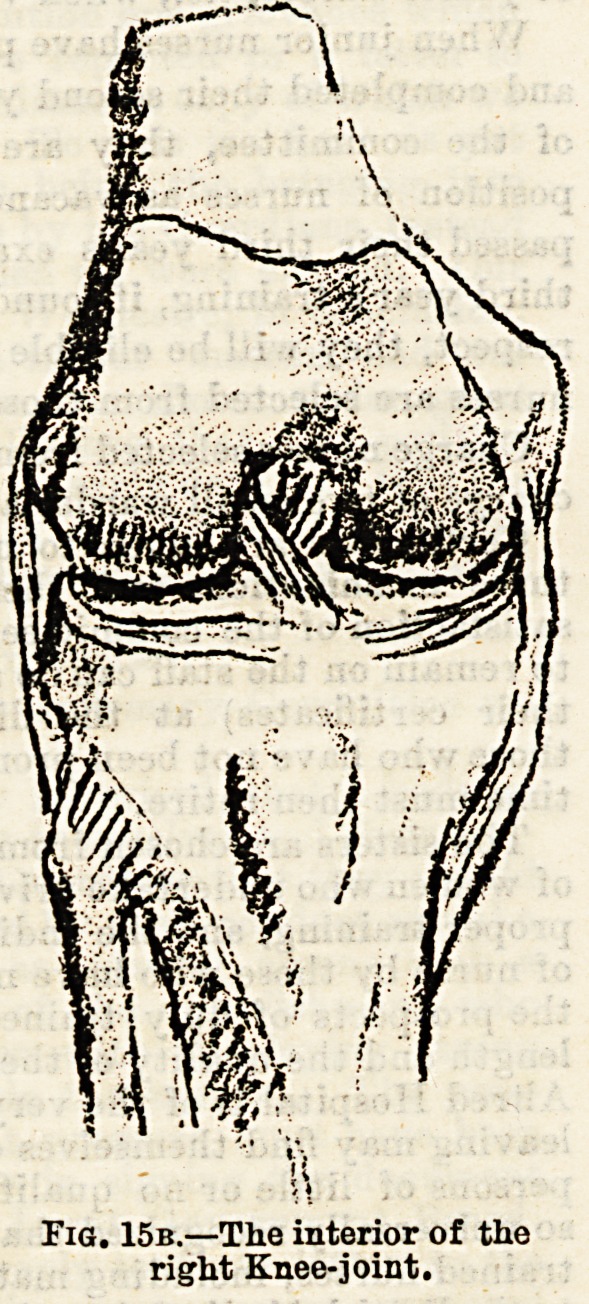


**Fig. 16. f4:**
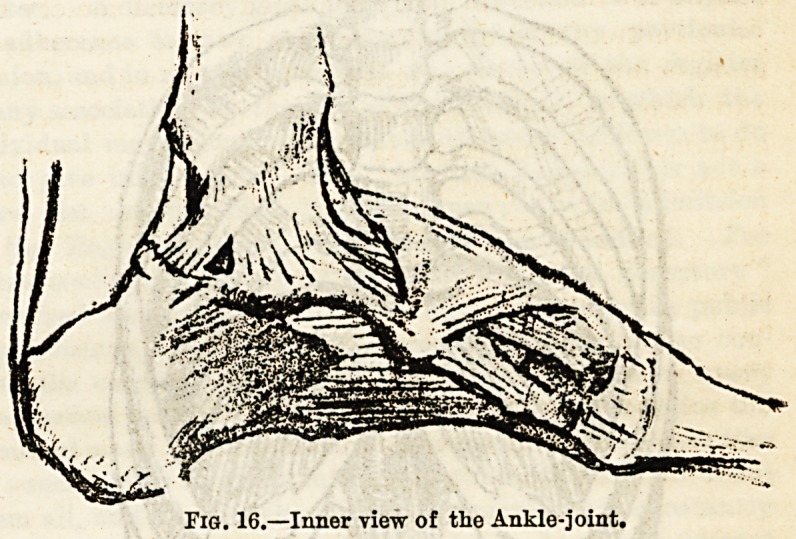


**Fig. 17. f5:**
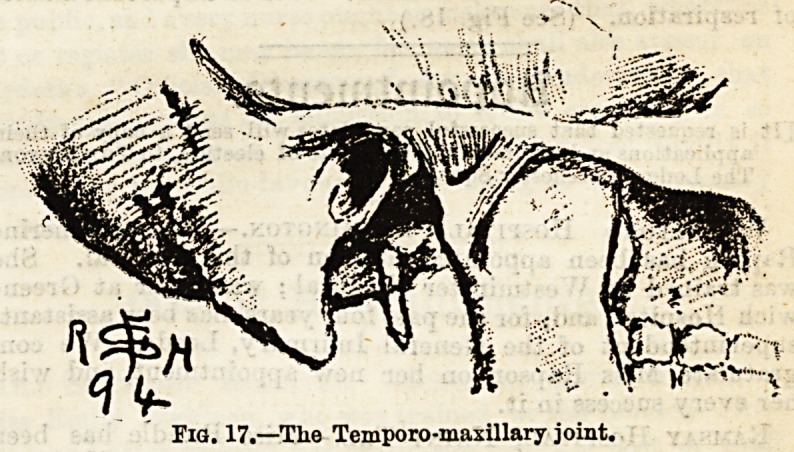


**Fig. 18. f6:**
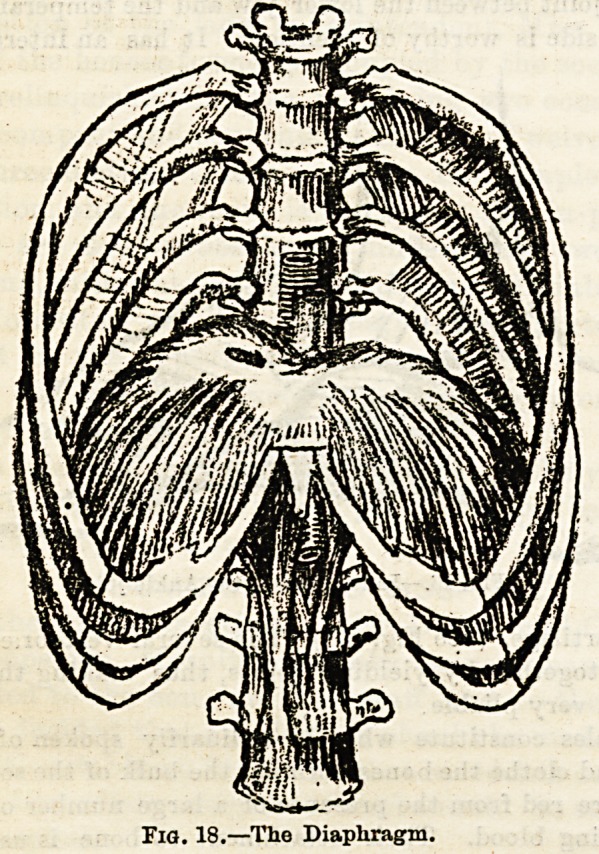


**Figure f7:**